# Multimodal regulation of myosin VI ensemble transport by cargo adaptor protein GIPC

**DOI:** 10.1016/j.jbc.2022.101688

**Published:** 2022-02-07

**Authors:** Ashim Rai, Rachit Shrivastava, Duha Vang, Michael Ritt, Fredrik Sadler, Shreyas Bhaban, Murti Salapaka, Sivaraj Sivaramakrishnan

**Affiliations:** 1 Department of Genetics, Cell Biology, and Development, University of Minnesota Twin Cities, Minneapolis, Minnesota, USA; 2 Department of Electrical and Computer Engineering, University of Minnesota Twin Cities, Minneapolis, Minnesota, USA

**Keywords:** adaptor protein, myosin, biophysics, endocytosis, FRET, membrane trafficking, optical tweezers, protein–protein interaction, structural biology, transport, AB, assay buffer, BSA, bovine serum albumin, Cam, calmodulin, CBD, cargo-binding domain, Dab2, disabled homolog 2, FL, full-length, FLASH, Fluorescin Arsenical Hairpin binder, GH2, glycoside hydrolase family 2, GIPC, Gα-interacting protein, C terminus, HMM, heavy meromyosin, IQ, isoleucine–glutamine, mCer, mCerulean, mCit, mCitrine, MIR, myosin-interacting region, MT, medial tail, NA, numerical aperture, PDF, probability distribution function, PT, proximal tail, Sf9, *Spodoptera frugiperda* 9, TCSPC, time-correlated single photon counting, TIRF, total internal reflection fluorescence

## Abstract

A range of cargo adaptor proteins are known to recruit cytoskeletal motors to distinct subcellular compartments. However, the structural impact of cargo recruitment on motor function is poorly understood. Here, we dissect the multimodal regulation of myosin VI activity through the cargo adaptor GAIP-interacting protein, C terminus (GIPC), whose overexpression with this motor in cancer enhances cell migration. Using a range of biophysical techniques, including motility assays, FRET-based conformational sensors, optical trapping, and DNA origami–based cargo scaffolds to probe the individual and ensemble properties of GIPC–myosin VI motility, we report that the GIPC myosin-interacting region (MIR) releases an autoinhibitory interaction within myosin VI. We show that the resulting conformational changes in the myosin lever arm, including the proximal tail domain, increase the flexibility of the adaptor–motor linkage, and that increased flexibility correlates with faster actomyosin association and dissociation rates. Taken together, the GIPC MIR–myosin VI interaction stimulates a twofold to threefold increase in ensemble cargo speed. Furthermore, the GIPC MIR–myosin VI ensembles yield similar cargo run lengths as forced processive myosin VI dimers. We conclude that the emergent behavior from these individual aspects of myosin regulation is the fast, processive, and smooth cargo transport on cellular actin networks. Our study delineates the multimodal regulation of myosin VI by the cargo adaptor GIPC, while highlighting linkage flexibility as a novel biophysical mechanism for modulating cellular cargo motility.

The adaptor GAIP-interacting protein, C terminus (GIPC) has been shown to recruit myosin VI to uncoated vesicles during endocytosis (1). The PDZ domain of GIPC interacts with cargo molecules, such as integrins and cell signaling receptors, whereas the glycoside hydrolase family 2 (GH2) domain has been shown to interact with myosin VI (2). Thus, by bridging the gap between the motor and proteins expressed on the cell surface, GIPC has been implicated as a key regulator of endocytosis for cellular signaling and adhesion molecules. Further supporting this role, GIPC overexpression has been found in numerous cancers (3). Interestingly, myosin VI has been correlatively shown to be overexpressed in the same cancers as GIPC, and downregulation of both GIPC and myosin VI was shown to ameliorate the cancerous phenotype (3). Therefore, understanding the regulation of myosin VI activity by GIPC is key to addressing these pathologies. Although a recent structural study has explored the receptor-mediated activation of GIPC–myosin VI interactions, the direct impact of GIPC on myosin VI motility is unknown and is the focus of this study (2).

Myosin VI is a multifunctional cytoskeletal motor with established roles in many cellular processes, including endocytosis, secretion, and the maintenance of stereocilia structure (4). Dysregulation of these processes has been implicated in numerous pathologies, including deafness and cancer (5, 6). Myosin VI is recruited to different subcellular compartments by distinct cargo adaptors, including disabled homolog 2 (Dab2) to clathrin-coated pits, GIPC to uncoated endosomes, target of Myb protein 1/2 to early endosomes, and lemur tyrosine kinase 2 to the endocytic recycling complex (7, 8). In parallel with its role in targeting myosin VI to distinct compartments, the structural impact of each adaptor in regulating motile behavior is poorly understood and addressed in this study.

The potential for individualized regulation of myosin VI function by distinct adaptors is mirrored by the multiple structural adaptations that have been reported for this motor (9). Myosin VI has a unique insert in its lever arm that reverses stroke direction and makes it the only known minus end–directed actin-based motor (10). Furthermore, the typical isoleucine–glutamine (IQ)-calmodulin (Cam) lever arm in myosin VI has two additions, an extensible three-helix bundle, termed the proximal tail (PT), and a single α-helical domain, termed the medial tail (MT). These lever arm additions increase stroke size and enable processive stepping of a forced truncated dimer of myosin VI (11, 12). In the absence of adaptor engagement, myosin VI exists as an autoinhibited monomer, facilitated by an intramolecular interaction between its catalytic and cargo-binding domains (CBDs) (11, 13). Cargo engagement or clustering on actin filaments has been reported to dimerize myosin VI, leading to processive motion on actin filaments (14). Finally, myosin VI also exhibits load-dependent anchoring to actin filaments, demonstrating the potential for cargo-mediated changes in actomyosin interaction kinetics (15). Despite this wealth of information on the structural features of myosin VI activity, it has mostly been obtained in the absence of cargo adaptors. Hence, the confluence of structural features to determine motility in different cellular contexts, facilitated by distinct adaptors, remains unclear. To address this challenge, we examine the intersection between GIPC engagement and each of the unique structural features of myosin VI and together their contribution to the motility of myosin VI ensembles.

In this study, we use a combination of motility assays, FRET-based conformational sensors, optical trapping, and DNA origami–based cargo scaffolds to dissect the multifunctional regulation of myosin VI by the myosin-interacting region (MIR) of GIPC. We report that GIPC MIR binding to myosin VI leads to an approximately twofold to threefold enhancement in myosin VI speed at an ensemble level. In contrast to other myosin VI adaptors like Dab2 and optineurin, GIPC MIR binding does not stimulate processive motility. At the structural level, GIPC MIR binding releases an autoregulatory interaction within myosin VI, leading to both extension and increased flexibility of the myosin VI lever arm. The enhanced flexibility leads to faster motor–actin interaction kinetics by reducing the dwell time of the motor on actin filaments. Taken together, these effects yield fast, smooth, and processive movements of GIPC-recruited myosin VI ensembles on cellular actin networks.

## Results

### GIPC enhances myosin VI speed in a motor density–independent manner

**Figure 1:**
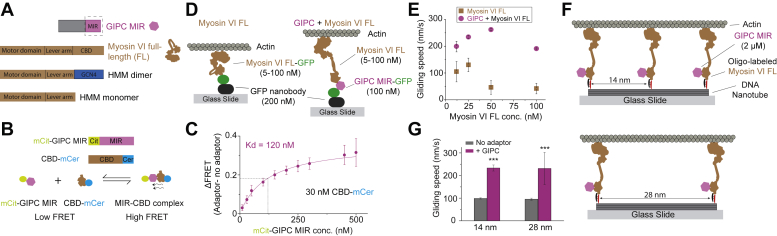
**GIPC MIR increases the actin gliding speed of myosin VI.***A*, schematic illustrating the cargo adaptors with MIR and myosin VI constructs used in this study. *B*, schematic representation of the construct and assay design for the bimolecular FRET assay. *C*, dose–response curve for the change in the FRET ratio (ΔFRET) *versus* the adaptor protein concentration for GIPC MIR. The ΔFRET is calculated as the difference between the FRET ratios for the adaptor present and no adaptor conditions. Each dose–response curve value was derived from three independent protein preparations. The dose–response curve was fit to a hyperbolic equation, and the dissociation constant (*K*_*d*_) was calculated as the protein concentration for the half-maximal response. Error bars are SD. *D*, schematic design of the myosin VI surface gliding assay. *E*, actin gliding speed with respect to myosin VI surface concentration in the presence and absence of GIPC. Error bars are SD. *F*, schematic design of myosin VI DNA nanotube actin gliding assay. *G*, actin gliding speed observed in the DNA nanotube actin gliding assay. For each condition, speed was computed from n ≥ 300 actin gliding traces. Error bars are SD. Significance was computed using Student’s *t* test. GIPC, GAIP-interacting protein, C terminus; MIR, myosin-interacting region.

**Figure 2:**
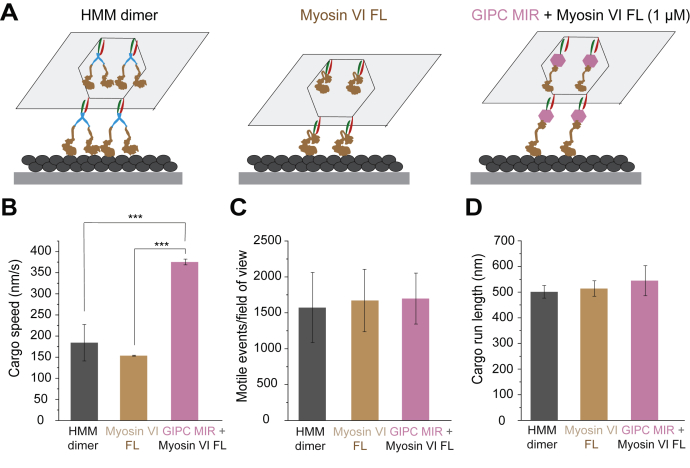
**Multimotor DNA origami motility on single actin filaments.***A*, schematic illustrating multiple motor DNA origami motility assays on single actin filaments. *B*–*D*, quantification of the average cargo speed (*B*), motile events per field of view (*C*), and cargo run length (*D*) of DNA origami motility for the different conditions listed. Mean value of all motile parameters for each experimental repeat was estimated from ≥50 DNA origami motility events. ∗∗∗*p* < 0.001, comparisons of nonsignificant values (*p* > 0.05) are not shown. All experimental conditions in the figure were performed across three independent protein preparations, and data shown represent the mean ± SD across preparations.

We first sought to characterize the effects of GIPC on the ensemble motility of myosin VI using *in vitro* motility assays. The myosin VI binding interface for GIPC has been previously well characterized (16). To minimize regulatory effects arising from nonmotor interacting domains of the adaptor, we used the minimal MIR of GIPC in this study (residues 261–333, Fig. 1*A*). We first estimated the binding affinity of the GIPC MIR and myosin VI interaction using a bimolecular FRET assay (Fig. 1*B*). A dose–response curve of the FRET change (ΔFRET), using a fixed concentration of mCerulean (mCer)-tagged myosin VI CBD and increasing concentrations of mCitrine (mCit)-tagged GIPC MIR, yielded a binding affinity (*K*_*d*_) of 120 nM (Fig. 1*C*). In all subsequent experiments, GIPC MIR, when free in solution, was used at a concentration >10 times higher than the *K*_*d*_ (2 μM) to ensure saturation binding to myosin VI. To minimize the influence of motor number on measured speeds, we used a DNA nanotube–based actin gliding assay (Fig. 1*D*). This assay has been previously used by us to precisely measure myosin V and myosin VI speed by patterning motors on a DNA nanotube with a well-defined spacing between motors to tightly control motor density (17). In addition to controlling motor density and nonspecific surface binding, the DNA nanotube assay geometry also allows us to use saturating concentrations of GIPC MIR (2 μM), allowing for a nonambiguous interpretation of the effect of adapter binding on myosin VI speed. We found that regardless of motor spacing on the DNA nanotube (14 *versus* 28 nm), GIPC MIR stimulated a 2.5-fold increase in actin gliding speed (Fig. 1*E*). To simulate multimotor-driven cargo complexes, full-length (FL) myosin VI, at saturating concentrations (1 μM), was recruited to GIPC MIRs patterned on DNA origami scaffolds (Fig. 2*A*). We compared GIPC MIR–recruited myosin VI with direct attachment of either myosin VI or a processive heavy meromyosin (HMM) dimer to the DNA origami scaffold. Recruitment of myosin VI through the GIPC MIR resulted in a 2.3-fold increase in scaffold speeds on single actin filaments (Fig. 2*B*). All three conditions had similar numbers of motile events per field of view (Fig. 2*C*) and had similar run lengths (Fig. 2*D*). Taken together, these data highlight the dramatic effect of GIPC MIR on the ensemble speeds of myosin VI-driven transport.

### Cargo processivity is driven by an ensemble of GIPC–myosin VI complexes

**Figure 3:**
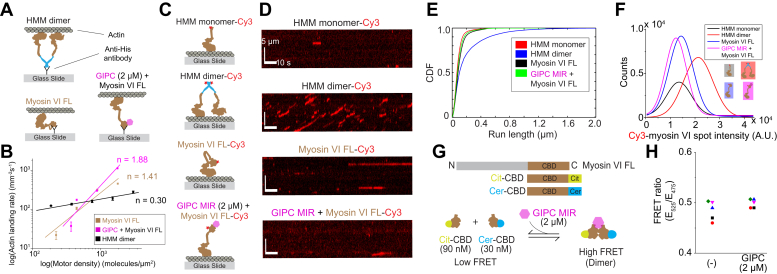
**Full-length (FL) myosin VI functions as a monomeric complex with GIPC MIR.***A*, schematic representation of myosin VI constructs and configuration used in the actin landing rate assay. *B*, log–log plot of actin landing rate with respect to motor density. A slope ≤1 indicates processive motility. *R*^2^ values for the linear fits were 0.913 for the HMM dimer, 0.884 for FL myosin VI, and 0.823 for FL myosin VI in the presence of GIPC. *C*, schematic indicating the myosin VI constructs used in the single-molecule TIRF assay. *D*, representative kymographs for the motility of the indicated constructs from *C*. *E*, cumulative distribution function (CDF) of the run lengths observed for each of the indicated constructs from *C* and *D*. *F*, Cy3 spot intensity for each of the constructs used in the single-molecule TIRF motility assay. *G*, schematic representation of the myosin VI cargo-binding domain (CBD) constructs and FRET assay used to probe GIPC MIR influence on myosin VI dimerization. *H*, FRET ratios of the constructs depicted in *G* in the presence or the absence of GIPC MIR. Experiments were performed using N ≥ 3 independent protein preparations. Significance was calculated using Student’s *t* test. GIPC, GAIP-interacting protein, C terminus; HMM, heavy meromyosin; MIR, myosin-interacting region; ns, not significant; TIRF, total internal reflection fluorescence.

Processive movement of myosin VI driven by homodimerization of the motor by adaptor proteins and membrane lipids has been a common theme of myosin VI regulation (14, 18, 19). Therefore, we wanted to explore the oligomerization state and processive behavior of myosin VI recruited through GIPC MIR. We hypothesized that given the role of GIPC in traffic of uncoated vesicles (20), it would facilitate processivity of myosin VI through dimerization. Ensemble processivity was first examined using an actin landing rate assay. Briefly, using the same geometry as in the surface actin gliding assay, varying concentrations of myosin VI are flowed onto the surface, and the rate of filament landing is monitored (Fig. 3*A*). The analysis of actin landing rate as a function of surface motor density has been conventionally used to infer motor processivity in gliding motility assays (21). A logarithmic plot of landing rate *versus* motor density fits to a line, and the slope of the linear fit (n) informs on the motor processivity. A slope >1 is indicative of nonprocessive motility, whereas a slope of ≤1 is a hallmark of processive motility. Using this assay, we found that recruitment of myosin VI through GIPC MIR did not make myosin VI processive (n = 1.88 *versus* n = 1.41 for myosin VI alone; Fig. 3*B*). In contrast, a constitutive HMM dimer of myosin VI was found to be processive using the same assay (n = 0.30). To address processive behavior of individual myosin VI molecules, we performed a single-molecule total internal reflection fluorescence (TIRF) motility assay of myosin VI in the presence or the absence of saturating amounts (2 μM) of GIPC MIR (Fig. 3*C*). As controls for a constitutively processive and nonprocessive myosin VI motor, we used a myosin VI HMM dimer and HMM monomer, respectively (Fig. 3*C*). Kymographs of single-molecule motility showed that myosin VI was nonprocessive, akin to the HMM monomer, and the addition of GIPC MIR did not alter the nonprocessive nature of myosin VI motility (Fig. 3*D*). In contrast, the HMM constitutive dimer of myosin VI displayed robust processive motility (Fig. 3*D*). Quantification of the single molecule run length further validated the observation that GIPC MIR binding did not alter the nonprocessive motility of myosin VI (Fig. 3*E*). To examine the oligomeric state of myosin VI in the single-molecule motility assay, we characterized the fluorescence intensity of single spots of Cy3-conjugated myosin VI (Fig. 3*F*). The spot intensity analysis was validated with HMM monomers and dimers. As expected, we observed an approximately twofold higher spot intensity in the HMM dimer as compared with the HMM monomer (Fig. 3*F*). The spot intensity of myosin VI was similarly distributed to that of the HMM monomer, and addition of saturating amounts of GIPC MIR did not alter myosin VI spot intensity distribution (Fig. 3*F*). To further investigate the oligomeric state of myosin VI bound to GIPC MIR, we designed a bimolecular FRET assay involving the myosin VI CBD (Fig. 3*G*). Analysis of the observed FRET ratios showed that addition of saturating amounts of GIPC MIR (2 μM) did not significantly increase CBD homodimerization (Fig. 3*H*). In summary, contrary to our initial hypothesis, binding of GIPC MIR neither facilitates oligomerization of myosin VI nor alters its nonprocessive motile behavior. Despite the lack of evidence for GIPC-mediated myosin VI dimerization, our data on monomeric myosin VI ensembles (Fig. 2) suggest that they are capable of processive cargo transport.

### GIPC MIR effects on myosin VI motility stem from the release of motor autoinhibition

**Figure 4:**
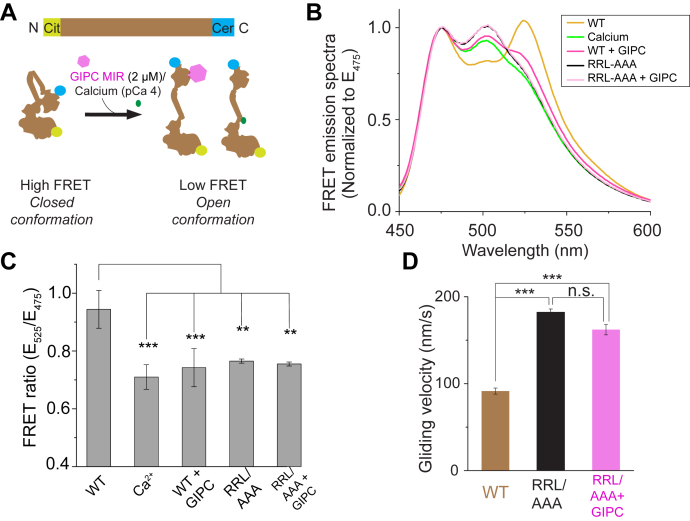
**GIPC MIR binding releases myosin VI autoinhibition.***A*, schematic illustrating the construct design and assay for the myosin VI conformational FRET sensor. *B*, representative emission spectra (normalized to emission at 475 nm) illustrating the occurrence of FRET between mCitrine and mCerulean in the myosin VI conformational sensor. Samples were excited with 430 nm light. *C*, FRET ratios calculated for the myosin VI FRET sensor under the indicated conditions for both the WT and RRL to AAA mutation in myosin VI. Significance was computed using one-way ANOVA with a post hoc Tukey’s test. *D*, gliding velocity of actin in a surface motility assay for the RRL to AAA mutation in myosin VI. Experiments were performed using N ≥ 3 independent protein preparations. ∗∗*p* < 0.01; ∗∗∗*p* < 0.001. GIPC, GAIP-interacting protein, C terminus; MIR, myosin-interacting region; ns, not significant.

A recurrent theme in the regulation of cytoskeletal motors is the release of the autoinhibitory interactions in a motor protein upon adaptor binding, thereby stimulating motility (22, 23, 24). Hence, we hypothesized that GIPC MIR binding may influence myosin VI autoinhibition, with concomitant effects on the lever arm. To test this hypothesis, we developed an intramolecular myosin VI conformational sensor, with an mCit and mCer FRET pair at the N and C terminus, respectively, of the FL myosin VI motor (Fig. 4*A*). A “closed” conformation should result in close proximity of the FRET probes to one another, resulting in a high FRET state. Conversely, a transition to the “open” conformation should separate the FRET probes, reducing the amount of FRET. Calcium was observed to decrease the FRET ratio in accordance with its ability to induce an open conformation (13). GIPC MIR binding caused an equivalent decrease in FRET ratio, indicating an open conformation (Fig. 4, *B* and *C*). To further examine the structural interface of the GIPC–myosin VI interaction, we mutagenized the putative GIPC-binding site, the RRL motif (18), in the CBD of a myosin VI conformational FRET sensor. Surprisingly, the RRL/AAA mutation led to a significant decrease in FRET, even without GIPC MIR addition (Fig. 4, *B* and *C*). Furthermore, addition of GIPC MIR to the RRL mutant FRET sensor did not lead to a further FRET change beyond the decrease seen in the mutant FRET sensor. This suggests that the putative GIPC-binding RRL motif also serves as the structural interface involved in maintaining the inactive closed conformation of the myosin VI motor. To test this idea, we examined the effect of the RRL/AAA mutation on the surface gliding velocities of actin filaments (Fig. 4*D*). The RRL/AAA mutation enhanced myosin VI speeds to levels similar to the effects of GIPC MIR on WT motors. GIPC MIR addition to the RRL mutant myosin VI had no additional effects on speed consistent with its binding to the RRL motif (Fig. 4*D*). Taken together, these findings support the competitive release of an autoinhibitory interaction within myosin VI as the primary mechanism for the enhanced motility driven by GIPC MIR.

### GIPC MIR increases stroke size and activity of myosin VI

**Figure 5:**
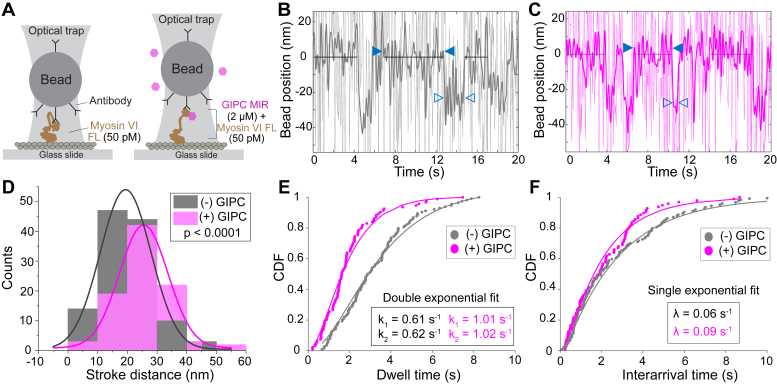
**GIPC MIR increases the stroke distance of the myosin VI lever arm.***A*, depiction of myosin VI labeled beads in a single-molecule optical trap experiment. *B* and *C*, example of optical trap traces for full-length myosin VI alone (*B*) and in the presence of GIPC MIR (*C*). *Black solid lines* indicate the baseline with *filled in arrowheads* indicating the interarrival time and *hollow arrowheads* indicating the dwell time. *D*, trap displacement data for myosin VI-labeled beads. *E* and *F*, cumulative distribution function (CDF) of the dwell (*E*) and interarrival (*F*) times for myosin VI in the presence and absence of GIPC MIR. Detailed explanations of the fits are presented in the Experimental procedures section. Experiments were performed using N ≥ 3 independent protein preparations. GIPC, GAIP-interacting protein, C terminus; MIR, myosin-interacting region.

Release of autoinhibition and structural changes in the myosin VI lever arm can impact the effective stroke size and consequently motor ensemble speeds. Hence, single-molecule optical trapping was used to quantify the effect of the GIPC MIR on myosin VI stroke size (Fig. 5*A*). The beads were labeled with low concentrations of motor to establish the single-molecule Poisson limit, with one to two out of 10 trapped beads showing motile events (see Experimental procedures section). Under these conditions, we observe single stroke events that were consistent with the monomeric nature of FL myosin VI (Fig. 5, *B* and *C*). We measured a stroke size of 19.3 ± 8.5 nm (Fig. 5*D*), consistent with previous optical trapping experiments for human FL myosin VI (25). The addition of saturating concentrations of GIPC MIR increases stroke size by 30% (Fig. 4*D*; 25.6 ± 8.1 nm; *p* < 0.0001). This modest change in myosin VI stroke does not address the approximately twofold increase in ensemble gliding speeds (Fig. 1). Hence, in parallel, we examined the effect of the GIPC MIR on the kinetics of the mechanochemical cycle of FL myosin VI. We quantified the dwell time and interarrival time of myosin VI-labeled beads in the optical tweezers set up (Fig. 5, *E* and *F*). Dwell time quantifies the duration of stroke events (*hollow blue arrowheads*; Fig. 5, *B* and *C*), whereas interarrival time quantifies the period between sequential stroke events (*solid blue arrowheads*; Fig. 4, *B* and *C*) in the optical trap. GIPC MIR decreases the dwell time of myosin VI on actin filaments from 0.83 to 0.5 s (Fig. 5*E*; *p* < 0.0001) and interarrival time between motile events from 14.8 to 11.1 s (Fig. 5*F*; *p* < 0.01). For high duty ratio motors such as a myosin VI, the dwell time on actin filaments is inherently rate limiting to the actomyosin crossbridge cycle (26). Hence, the 1.6-fold decrease in dwell time combined with a 1.3-fold increase in stroke size translates into an approximately twofold increase in speed at the actomyosin crossbridge that sufficiently captures the approximately twofold to threefold increase in gliding speeds observed in motility assays (Fig. 1*E*).

### GIPC MIR–mediated effects on cargo speeds stem from changes in stiffness of motor–cargo linkage

**Figure 6:**
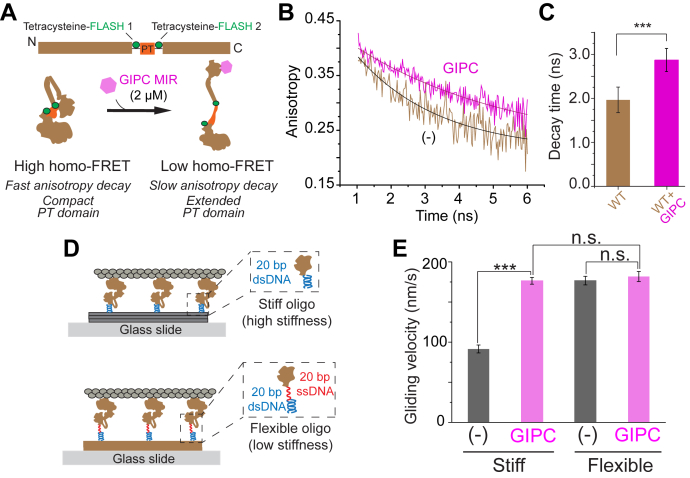
**GIPC MIR decreases actomyosin crossbridge stiffness, leading to enhanced motor speed.***A*, schematic of the design of the proximal tail conformation sensor for myosin VI. *B*, representative anisotropy decay traces for the proximal tail conformation sensor in the presence and absence of GIPC MIR. *C*, decay times for the proximal tail conformation sensor for either the WT or RRL to AAA mutant in both the presence and absence of GIPC MIR. *D*, schematic depiction of the surface gliding nanotube assay with stiff and flexible linkages. *E*, actin gliding velocities of myosin VI in the presence or absence of GIPC MIR, when attached to nanotubes *via* either a stiff or a flexible oligo. Experiments were performed using N ≥ 3 independent protein preparations. Significance was assessed using either Student’s *t* test or a two-way ANOVA with post hoc Tukey’s test. ∗∗∗*p* < 0.001. GIPC, GAIP-interacting protein, C terminus; MIR, myosin-interacting region; ns, not significant.

We have previously used synthetic DNA linkers between motor and scaffolds to demonstrate that motor–cargo linkage stiffness can tune actin gliding speeds (17). Hence, we hypothesized that release in myosin VI autoinhibition by GIPC MIR could enhance the mechanical flexibility of the motor–cargo linkage, thereby providing an elegant biophysical mechanism for the twofold to threefold enhanced cargo speeds. Supporting this concept, activation of myosin VI through cargo binding has been implicated in structural changes in the lever arm of the motor (11). Of particular interest is the PT domain that consists of a three-helix bundle that unfolds upon motor activation to extend the length and the flexibility of the myosin VI lever arm (12). Hence, we developed a homo-FRET–based PT conformation sensor, by introducing tetracysteine sites labeled with the biarsenical fluorescent dye, FlAsH-EDT_2_ (FLASH; Fluorescin Arsenical Hairpin binder-Ethanedithiol), flanking the PT domain of myosin VI (Fig. 6*A*). The use of a homo-FRET approach was necessitated by the ease of protein labeling with a single fluorescent dye that allowed precise control over saturable labeling of both sites. The radiative energy transfer during homo-FRET is a reversible process that leads to no change in the overall emission spectrum and fluorescence lifetime. Hence, a simple fluorescent lifetime measurement could not be used. However, the dipole orientation of the homo-FRET donor and acceptor can differ and the fluorescence emission from FRET acceptor with consequent differences in the polarization of the emission from donor and acceptor. This effect can be captured by measuring the time-resolved fluorescence anisotropy of the sensor. We expect that a folded PT domain would result in the close proximity of labeled fluorescent dyes (∼3 nm; (12)), albeit without a significant correlation between their dipole orientations. The resulting resonance energy transfer between the donor and acceptor would then result in a rapid decay in fluorescence anisotropy. Extension of the PT domain, in accordance with a previous study (12), would decrease resonance energy transfer and therefore reduce the decay in anisotropy over time. The addition of GIPC MIR to the PT conformational sensor, at saturating concentrations (2 μM), significantly slows the anisotropy decay (Figs. 6, *B* and *C* and S1) and is consistent with GIPC promoting an open conformation of the PT domain. A single tetracysteine site control did not display anisotropy decay changes upon GIPC MIR binding, indicating that the observed changes in homo-FRET were not simply a result of intermolecular interactions or changes in rotational diffusion of myosin VI resulting from GIPC MIR binding (Fig. S1, *E* and *F*).

The open conformation adopted by myosin VI bound to GIPC MIR (Fig. 4*C*), combined with its effects on the myosin lever arm (Fig. 6*C*), suggests that GIPC MIR can alter the mechanical properties of the actomyosin crossbridge. We have previously shown that stiff and flexible DNA attachment strategies can be used to tune actin gliding speeds, when myosins are precisely patterned on DNA nanotubes (17). Specifically, flexible attachment of forced dimers of myosin V and VI to DNA nanotubes substantially increased actin gliding speeds in motility assays (17). Using this strategy to vary motor–cargo linkage stiffness with FL myosin VI, we find an approximately twofold increase in actin gliding speeds, a similar extent as observed with GIPC MIR (Fig. 6, *D* and *E*). It must be acknowledged that the magnitude of the effect of GIPC MIR on the actin gliding speed of FL myosin VI is variable (Figs. 1*E* *versus* 6*E*). Each dataset represents three independent protein preparations, whereas the preparations and assay conditions do not overlap between these two datasets, and the variability likely reflects heterogeneity stemming from recombinant protein preparations. Nonetheless, the addition of GIPC MIR in the presence of the flexible linkage had no additional effect (Fig. 6*E*). Previous modeling from our laboratory showed a nonlinear relationship between stiffness and gliding speed, such that the gains in speed eventually plateau for low linkage stiffness (17). Hence, our observations strongly support the reduced linkage stiffness precipitated by GIPC, as an underlying mechanism to enhance cargo speeds.

### GIPC mediates smooth and processive movements of myosin VI cargo on a cellular actin network

**Figure 7:**
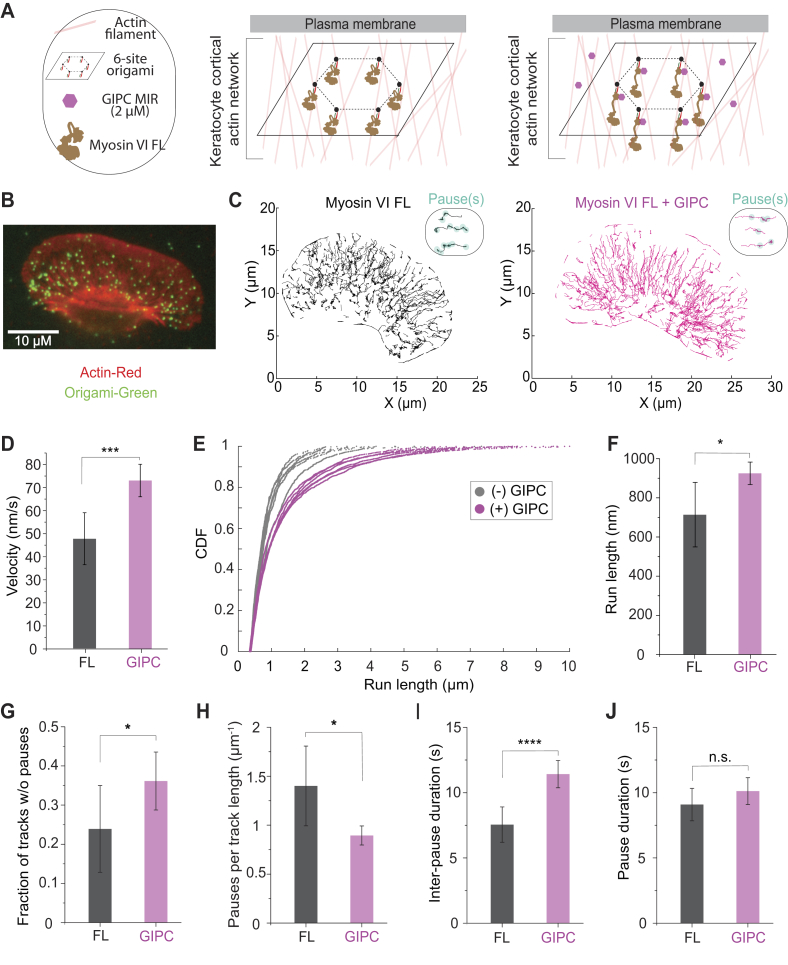
**GIPC MIR drives smooth and processive ensemble cargo motility.***A*, schematic illustrating the configuration of myosin VI on the DNA origami scaffold. *B*, example of merged image of the actin cytoskeleton extracted from a fish keratocyte (*red*) with DNA origami (*green*). The scale bar represents 10 μm. *C*, representative trajectories of myosin VI-labeled DNA origami on a keratocyte actin network. A sampling of trajectories is shown to aid in visualization of behavior. *Inset*, representative trajectories depicting pausing behavior (*cyan circles*). *Circles* represent one or more pauses in close proximity. *D*, average velocity of DNA origami scaffold with full-length myosin VI, in the presence or the absence of GIPC MIR, on the keratocyte actin network. *E*, individual cumulative distribution functions (CDFs) of run lengths for six measured keratocytes. *F*, trajectory run length in the absence or the presence of GIPC MIR. *F*, run lengths were calculated as the average run length of trajectories greater than 320 nm for each keratocyte. The data shown are the mean value for six keratocytes from each condition. *G*–*J*, analysis of parameters involved in pausing behavior of DNA origami scaffolds on the actin cytoskeletal network. *G*, fraction of analyzed tracks (run length >320 nm) without pauses. *H*, the number of pauses per micron distance traveled by each scaffold. *I*, duration between pauses. *J*, the duration of each pause. *G*–*J*, six keratocytes were analyzed per condition using protein from at least two separate protein preps. Significance was calculated using Student’s *t* test. ∗*p* < 0.05; ∗∗∗*p* < 0.001; ∗∗∗∗*p* < 0.0001; ns. GIPC, GAIP-interacting protein, C terminus; MIR, myosin-interacting region; ns, not significant.

GIPC MIR binding has several distinct effects on myosin VI structure and function, including release of autoinhibition (Fig. 3), changes in lever arm conformation (Fig. 6), and stroke size (Fig. 5), while maintaining monomeric interactions (Fig. 2). To understand how these effects contribute to the motile behavior of myosin VI ensembles, we used DNA origami scaffolds patterned with multiple GIPC MIR–myosin VI complexes on both single actin filaments (Fig. 2) and a cellular actin network (Fig. 7). Previous studies have shown that multiple monomeric myosin VI motors can still drive processive cargo motility (27). Therefore, we contrasted the run length of DNA origami scaffolds patterned with four FL myosin VI motors, in the presence or the absence of GIPC MIR, with forced HMM dimers (Fig. 2*A*). FL myosin VI motile events and run lengths were indistinguishable from those driven by HMM dimers, supporting processive behavior of cargo scaffolds despite the lack of individual motor processivity (Fig. 2, *C* and *D*). Addition of GIPC MIR did not substantially augment either the number of motile events or scaffold run lengths but did significantly increase cargo speeds (Fig. 2*B*). Thus, at the ensemble level, multiple GIPC–myosin VI complexes provide processive cargo movement at enhanced speeds. To examine cargo behavior on cellular actin networks, we leveraged detergent-extracted and phalloidin-stabilized lamellipodia from fish epidermal keratocytes (27) (Fig. 7). The origami scaffolds used in this experiment were patterned at six sites with FL myosin VI and examined in either the presence or the absence of GIPC MIR (Fig. 7, *A*–*C*). Consistent with single filament observations, the presence of GIPC MIR significantly enhanced cargo speeds (Fig. 7*D*). In contrast to data from single filaments, the presence of GIPC MIR enhanced cargo run lengths (Figs. 2*C* and 7, *E* and *F*). We speculate that a combination of increased motor number (six myosin VI–binding sites on the DNA origami in our keratocyte assays compared with four sites for single filament measurements) combined with myosin access to multiple actin filaments provide more opportunities for motors to bind and sustain cargo movement. Closer examination of cargo movement revealed that a greater fraction of trajectories lacked pauses in the presence of GIPC MIR (>100 nm mean square displacement for at least 4 s; Fig. 7, *C* and *G*). Furthermore, GIPC MIR caused fewer pauses per unit trajectory length (Fig. 7*H*) with less frequent pausing (Fig. 7*I*). Interestingly, the pause events lasted the same duration whether GIPC MIR was present or not (Fig. 7*J*), indicating that the pausing behavior stems from a lack of coordination of the motors during motion rather than on the ability of the adaptor to recover motility after the cargo had stopped. Taken together, these studies demonstrate that GIPC–myosin VI ensembles display fast, smooth, and processive movements on cellular actin networks.

## Discussion

**Figure 8:**
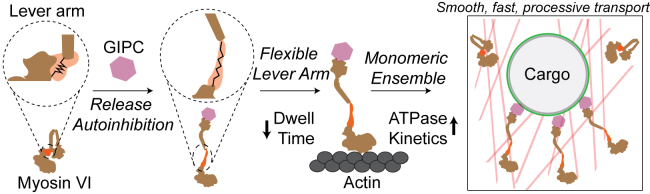
**Model for multimodal regulation of the dual transporter-anchor function of myosin VI by GIPC.** Myosin VI binding to GIPC MIR releases motor autoinhibition, extends the lever arm, and increases the flexibility of the motor–cargo linkage. This results in increased speed of single motors and enhances the processivity in ensembles. GIPC-mediated regulation leads to fast and smooth cargo transport across dense actin networks. GIPC, GAIP-interacting protein, C terminus; MIR, myosin-interacting region.

Our study highlights the integrated effects of multiple modes of adaptor-mediated regulation of the cytoskeletal motor myosin VI (Fig. 8). GIPC MIR binding results in the release of autoinhibitory interactions within myosin VI. This allows for conformational changes in the lever arm, in particular the PT, thereby increasing both myosin stroke and flexibility of the crossbridge formed by the myosin, between the actin and the cargo. A flexible link enables faster dissociation and association of the actomyosin crossbridge, which combined with the increased myosin stroke translates to approximately twofold to threefold greater speeds of myosin VI ensembles. Although GIPC MIR was not observed to affect the oligomerization of the motor, ensembles of myosin VI–GIPC MIR complexes were capable of similar run lengths as ensembles of forced processive HMM dimers. The individual modes of myosin VI regulation merge to allow for fast, smooth, and processive cargo transports on cellular actin networks. Taken together, our in-depth characterization of this motor–adaptor interaction and interplay among different regulatory modes delineates motor–cargo linkage stiffness as a novel mechanism of cytoskeletal motor regulation used to specifically direct traffic within the cell.

Here, we demonstrate the ability of GIPC MIR binding to relieve the autoinhibited conformation of myosin VI. Autoinhibition of cytoskeletal motors is one of the most prevalent and well-studied themes of motor regulation and widely believed to prevent futile ATP hydrolysis when the motor is not engaged to a cellular cargo (22, 23, 24). This autoinhibited conformation typically occurs through an interaction between the cargo-binding tail domain and the ATP-hydrolyzing motor domain that results in a folded conformation and a decrease in ATPase activity of the motor. Conversely, binding to a cargo and/or divalent cations has been shown to relieve the autoinhibited motor conformation, thereby activating the motor and stimulating ATPase activity. A structurally well-characterized unconventional myosin, myosin Va, presents a prototypical example of a head–tail autoinhibited motor, where binding to the cargo adaptor melanophilin was shown to relieve the autoinhibited conformation (28, 29). For myosin VI, Batters *et al.* (13) used single-particle EM analysis to show that myosin VI existed in a folded back and autoinhibited conformation, and calcium binding resulted in a switch to a partially open motor conformation. Similarly, using an intramolecular FRET sensor, Fili *et al.* (30) showed that binding to the cargo adaptor nuclear dot protein 52 kDa relieved the folded back conformation of myosin VI. In this study, we used an intramolecular FRET sensor to probe the effect of GIPC MIR binding on the autoinhibited conformation of myosin VI. We found that, much like the adaptors outlined previously, GIPC MIR binding was sufficient to switch myosin VI from an autoinhibited to an open conformation. Furthermore, our results show that the GIPC-binding RRL motif on the myosin VI CBD is itself involved in maintaining the head–tail autoinhibition of myosin VI. Thus, our results highlight an elegant mechanism, wherein sequestration of the RRL motif by GIPC MIR breaks the head–tail interaction to relieve motor autoinhibition.

Myosin VI is unique among unconventional myosins in having only a single Cam-binding IQ motif, suggesting that it has a short lever arm and by consequence a short step size on the actin filament (9). However, a wealth of studies have revealed that myosin VI has additional domains in its tail region, termed the PT and medial tail (MT), which contribute to an extension of the “effective” myosin VI lever arm, thereby extending its reach to take a longer step. The myosin VI PT domain is a three-helix bundle that exists in a folded conformation in the autoinhibited and inactive myosin VI (11, 12). Whereas previous studies had demonstrated that the PT domain can unfold to increase myosin VI step size, they had done so in the context of a truncated and artificially homodimerized motor (11, 12). Here, we show that GIPC MIR binding to the FL myosin VI unfolds the PT domain, which in concert with an extended MT domain can increase the myosin VI stroke. Our results, therefore, provide a direct structure–function connection for adaptor-mediated conformational changes in the lever arm that enhance the motile properties of myosin VI.

Linkage stiffness has been previously stipulated as a mechanism to influence the motility of cytoskeletal motor ensembles (31, 32). Artificially dimerized myosin VI ensembles patterned on DNA nanotubes yielded higher actin gliding velocities when the stiffness of the synthetic linkage between motor and nanostructure was decreased (17). Likewise, artificially dimerized myosin VI motors patterned on DNA origami scaffolds moved faster on single actin filaments when the motor–cargo linkage stiffness was reduced (32). These studies demonstrated a biophysical mechanism to modulate cargo speed, whereas its cellular relevance has not been established. The motor–cargo linkage can be considered as a serial combination of two harmonic springs, the motor and the cargo adaptor. The net mechanical stiffness of a serial combination of harmonic springs is dictated by the most flexible structural element in this combination. The mechanical stiffness of the motor itself is a function of the structural conformation of the motor. In its autoinhibited state, myosin VI exhibits a more compact conformation (11), whereas release of autoinhibition by GIPC MIR exposes more flexible elements of the myosin VI lever arm, specifically the PT and MT domains. Furthermore, the conformational changes in the PT, measured using anisotropy, are consistent with previous reports of the unfolding of this domain during myosin VI stepping (12). Hence, we propose that the release of myosin VI autoinhibition enhances motor flexibility and consequently the net flexibility of the motor–cargo linkage. In turn, the increased flexibility of the motor–cargo linkage has been previously shown to decrease the motor dwell time on the actin filament (*t*_dwell_) (32). Here, we observe a similar effect of GIPC MIR binding on single molecule *t*_dwell_ (Fig. 5*E*). Because *t*_dwell_ is a rate-limiting factor in the actomyosin ATPase cycle for myosin VI, decreasing it enhances crossbridge turnover rates, with consequent higher cargo speeds. Taken together, these observations demonstrate that tuning myosin VI flexibility by the release of autoinhibition is the underlying basis of the enhanced cargo speeds stimulated by GIPC MIR.

The significance of myosin VI homodimerization in cargo processivity is unclear. Earlier studies had predicted that myosin VI has an intrinsic potential for dimerization through a putative coiled-coil motif in its tail domain (33). However, multiple subsequent studies have shown that FL myosin VI is natively monomeric and requires binding to cargo proteins and/or lipids to assist dimerization and consequent processivity (11). Likewise clustering of myosin VI monomers on actin filaments, presumably because of a high local concentration facilitated by adaptor/lipid membrane engagement, has also been shown to induce processive movement (34). Spudich *et al.* (18) showed that binding to phosphatidylinositol 4,5-bisphosphate phospholipids induces an increase in helicity in the myosin VI CBD that results in motor dimerization. Similarly, structural studies showed that cargo adaptor proteins such as Dab2 and optineurin were capable of dimerizing myosin VI and driving processive motility (14, 19). In contrast, multiple truncated myosin VI monomers recruited to an artificial polystyrene bead scaffold were sufficient to drive processive motility on a cellular actin network (27). In parallel, structures of GIPC MIR in complex with the CBD of myosin VI support a 1:1 molar ratio for this interaction (2). However, crystal packing and analytical ultracentrifugation analysis suggest oligomerization of the GIPC MIR and CBD in a 5:5 complex (2). Furthermore, FL GIPC exists as a domain-swapped dimer, wherein the MIR within the GH2 domain is obscured by binding in *trans* with the PDZ domain. Interaction of GIPC with a signaling receptor such as plexin D1 releases the domain-swapped dimer and enables interaction with myosin VI. This activated GIPC is suggested to oligomerize through intermolecular interactions involving a GH1 domain. Together, these data suggest that the GIPC-mediated clustering of myosin VI could facilitate membrane traffic. Accordingly, our studies with myosin VI ensembles on DNA origami scaffolds, interacting with single actin filaments (Fig. 2) and cellular actin networks (Fig. 7), sufficiently demonstrate robust cargo motility.

Our study intentionally used the MIR, rather than the FL GIPC, to focus on the adaptor–myosin VI interaction. Access to the GIPC MIR, however, is regulated by the binding of the GIPC PDZ domain to a cell surface receptor (2). Such regulation of adaptor–motor engagement can prevent motor mislocalization and provide spatiotemporal control of motor activity. A number of cell signaling receptors, including plexin D1, β1-adrenergic receptor, megalin, glucose transporter 1, and integrin 5a, engage GIPC through this PDZ domain (35, 36). Hence, recruitment of GIPC to these receptors could enhance the motile properties of myosin VI cargo ensembles as outlined in this study. We propose that such super-regulation of motor ensembles can influence membrane trafficking and signaling outcomes for these receptors.

## Experimental procedures

### List of constructs

GIPC MIR: GH2 domain of human GIPC1 (amino acids 261–333) was used as the minimal myosin VI interaction region as defined previously (2).

Myosin VI FL: The isoform of human myosin VI containing both the long insert and short insert was used in all experiments involving the FL myosin VI.

C-terminal GFP-tagged and SNAP-tagged fusion proteins of adaptor MIRs were cloned in the insect cell expression vector pBiex1 (Novagen) with a C-terminal FLAG tag incorporated to facilitate affinity-based protein purification. An N-terminal mCit version of adaptor MIRs with a FLAG tag was used for bimolecular FRET assays. A GFP nanobody construct with C-terminal SNAP and FLAG tags in pBiex1 vector was used to bind GFP-tagged proteins as reported by us previously (17, 37, 38).

The following versions of myosin VI were used in this study:

1. Myosin VI FL-GFP: A C-terminal GFP-tagged and FLAG-tagged construct of FL human myosin VI cloned in pBiex1.
2. Myosin VI FL-FLAG: A C-terminal FLAG-tagged version of FL human myosin VI cloned in pBiex1.
3. Myosin VI FL-SNAP: A C-terminal SNAP-tagged, FLAG-tagged, and a 6×-His-tagged construct of FL human myosin VI cloned in pBiex1.
4. Myosin VI ΔCBD dimer: Myosin VI, residues 1–992 from *Sus scrofa*, containing both the IQ and SAH domains with a GCN4 leucine zipper (for dimerization), a SNAP tag (for DNA oligo attachment), a FLAG tag (for purification), and a 6×-His tag cloned in pBiex1.
5. Myosin VI ΔCBD monomer: Myosin VI, residues 1–992 from *Sus scrofa*, containing both the IQ and SAH domains with a SNAP tag, FLAG tag, and a 6×-His tag cloned in pBiex1.
6. Myosin VI conformational FRET sensor: Human myosin VI FL with an N-terminal mCer and a C-terminal mCit fluorescent tag with a FLAG tag and a 6×-His tag cloned in pBiex1.
7. Myosin VI PT domain sensor: Human myosin VI FL with tetracysteine motifs inserted after lysine 833 and lysine 921 flanking the PT domain with a C-terminal FLAG tag cloned in pBiex1. A similar construct with a single tetracysteine inserted after lysine 833 was used as a control construct.
8. Myosin VI CBD constructs: The C-terminal amino acids 1030–1284 of human myosin VI was used as the putative CBD of myosin VI as defined previously. C-terminal mCer-tagged versions of CBD with a FLAG tag cloned in pBiex1 were used in this study.

### Buffers and reagents

Assay buffer (AB): 20 mM imidazole (pH 7.5), 25 mM KCl, 4 mM MgCl_2_, 1 mM EGTA, 1 mM DTT. AB.bovine serum albumin (BSA): AB containing 1 mg/ml BSA.

AB.BSA.Cam: AB.BSA containing 10 μM Cam. AB.BSA.nt: AB.BSA containing 0.1 μM random nucleotide mix.

Stop solution (for ATPase assay): 60 mM EDTA (pH 6.5), 6.6% SDS.

Developing solution (for ATPase assay): 0.5% ammonium molybdate (2% stock in 4 N H_2_SO_4_), 5 mg/ml ferrous sulfate.

Lysis buffer (for protein purification): 20 mM imidazole (pH 7.5), 200 mM NaCl, 4 mM MgCl_2_, 0.5 mM EDTA, 1 mM EGTA, 0.5% IGEPAL CA-630, and 7% sucrose

Wash buffer (for protein purification): 20 mM imidazole (pH 7.5), 150 mM KCl, 5 mM MgCl_2_, 1 mM EDTA, and 1 mM EGTA

### Protein expression and purification

All proteins used in this study were expressed and purified in *Spodoptera frugiperda* 9 (Sf9) insect cells and purified using a FLAG tag–based affinity purification. The protocol is described briefly here. Transient transfection of constructs in Sf9 cells was achieved using the Escort IV system (MilliporeSigma). For protein purification, transiently transfected Sf9 cells at a cell number of ∼60 × 10^6^ cells were centrifuged at 350*g* for 5 min to pellet the cells. The supernatant was discarded, and the cell pellet was resuspended in 3 ml of ice-cold lysis buffer supplemented with 1 μg/ml PMSF, 10 μg/ml aprotinin, and 10 μg/ml leupeptin. Cell lysis was achieved by 20 cycles of pipetting of the resuspended cell pellet. The cell lysate was centrifuged at 176,000*g* for 25 min at 4 °C in a TLA 100.4 rotor (Beckman) to pellet the cell debris. The supernatant was incubated with 50 μl of anti-FLAG M2 affinity resin for 2 h at 4 °C with rotation. The resin–lysate mix was centrifuged at 1000*g* for 1 min at 4 °C to pellet the resin. The resin was then washed thrice with ice-cold wash buffer supplemented with 1 μg/ml PMSF, 10 μg/ml aprotinin, and 10 μg/ml leupeptin by resuspending and then pelleting the resin by centrifuging at 1000*g* for 1 min at 4 °C. The supernatant from the last wash was removed, and the resin was resuspended in wash buffer supplemented with 0.2 mg/ml FLAG peptide (MilliporeSigma) to elute the protein. Protein estimation was carried out by either using a NanoDrop spectrophotometer for fluorescently tagged proteins or using BSA standards on a 10% SDS-PAGE gel for nonfluorescent proteins.

### Conjugation of oligo with benzylguanine ester

Oligo with a 5′ amino modification (AmMC6; IDT) at a final concentration of 168 μM was mixed with benzylguanine *N*-hydroxysuccinimide ester (NEB) at a concentration of 11.6 mM in 100 mM sodium borate buffer (pH 8.5) and incubated at 37 °C for 4 h with rotation. Labeled oligo was then purified using Illustra G-50 micro columns (GE Healthcare) twice, and the concentration of oligo was estimated using a NanoDrop spectrophotometer.

### Oligo labeling of SNAP-tagged proteins for DNA nanotube and DNA scaffold assays

Protein purification was carried out in a similar way till the incubation of cell lysate on the anti-FLAG resin and three washes of the resin with wash buffer. Then the resin was resuspended in 200 μl of wash buffer, and benzylguanine-labeled oligo was added at a final concentration of 1.5 μM. The labeling reaction was then incubated at 4 °C overnight with rotation. After incubation, the resin was washed thrice with wash buffer to remove the excess oligo, and elution using FLAG peptide was carried out as described previously.

### FLASH labeling of tetracysteine sites in myosin VI PT domain sensor

The protocol for FLASH labeling of tetracysteine-tagged myosin VI PT domain sensor was similar to that used for oligolabeling of SNAP-tagged proteins. The biarsenical fluorescent ligand FLASH (Cayman Chemical Company) was added to the FLAG resin-bound protein at a final concentration of 2 μM, and labeling was performed overnight at 4 °C with rotation. The unbound excess FLASH ligand was removed with three washes with wash buffer, and the protein was eluted using FLAG peptide. As a control for nonspecific effects, a single tetracysteine site flanking the PT domain was used (Fig. S1*A*). Stepwise photobleaching, motility and ATPase assays were used to characterize fluorophore labeling and functionality (Fig. S1, *B*–*D*). We observe single-step and two-step photobleaching events for the control and two-site sensors, consistent with the number of tetracysteine labeling sites (Fig. S1, *B* and *C*). The two-site sensor demonstrates decreased motility consistent with a lower ATPase rate that likely reflects the influence of the fluorophore on the chemomechanical cycle (Fig. S1*D*).

### Surface actin gliding motility assay

Plasma cleaned glass coverslips (22 × 22 mm; Corning) were coated with 0.1% colloidin (Electron Microscopy Sciences) in amyl acetate. Flow chambers were prepared by sticking the colloidin-coated coverslips to a glass slide using strips of double-sided tape. GFP nanobody was added to the flow chamber at a final concentration of 200 nM in AB for nonspecific surface adsorption by incubation for 4 min at room temperature. The unbound GFP nanobody was washed off with three washes of AB. Surface passivation was then carried out by AB.BSA incubation for 4 min. Then, the GFP-tagged protein (adaptor MIR or myosin VI FL) at a concentration of 200 nM was added to the chamber and incubated for 4 min. The unbound protein was washed by three washes with AB.BSA. For adaptor MIR-based assay, myosin VI FL-FLAG was added to the chamber at a concentration of 200 nM and incubated for 4 min. The unbound myosin VI was washed with AB.BSA. Finally, a motility mix was prepared in AB.BSA.Cam buffer containing Alexa-647 phalloidin (Invitrogen)–labeled F-actin at 0.5 μM, an ATP regenerating mix (1 mM phosphocreatine and 0.1 mg/ml creatine phosphokinase), an oxygen-scavenging system (0.6% glucose, 45 μg ml^−1^ catalase, and 25 μg ml^−1^ glucose oxidase), and 2 mM ATP and added to the flow chamber. The actin gliding motility was assayed using a Nikon Eclipse Ti inverted epifluorescence microscope at 100× using a 1.4 numerical aperture (NA) oil immersion objective at a frame rate of 1 Hz for 2 min. The actin gliding data were analyzed using FIESTA software (39). For landing rate experiments, the myosin VI concentration was varied from 200 nM to the low dilution at which actin landing on the surface could no longer be observed. Surface density of motors and actin landing rates were calculated as described previously (40).

### Nanotube actin gliding motility assay

The detailed protocol for preparing DNA nanotubes has been described by us previously (17). Flow chambers were prepared similar to that described under surface motility assay. BSA conjugated with biotin was added to the flow chamber at 1 mg/ml in AB and incubated for 4 min. Unbound BSA–biotin washes with three flows of AB and AB.BSA were added to the chamber for surface passivation for 4 min. Neutravidin at 0.2 mg/ml in AB.BSA was added to the flow chamber and incubated for 4 min. Unbound neutravidin was removed with three flows of AB.BSA. DNA nanotubes with a biotin strand for surface attachment were added to the flow chamber at a concentration of 50 nM in AB.BSA.nt buffer and incubated for 4 min. Unbound nanotubes were washed with three flows of AB.BSA.nt. Myosin VI FL-SNAP conjugated with the attachment oligo was added to the flow chamber at 100 nM in AB.BSA.nt and incubated for 4 min to saturably label the nanotubes. Unbound myosin VI was removed by three washes with AB.BSA.nt. Adaptor-MIR at a concentration of five and greater times the *K*_*d*_ of adaptor–myosin VI interaction was added for binding in *trans* to the nanotube-bound myosin VI and incubated for 4 min. Alexa-647 phalloidin–labeled F-actin was sheared by three passes through a Hamilton syringe, and a motility mix similar to that of the surface motility assay in AB.BSA.nt.Cam buffer was added to the flow chamber. Motility was assayed and analyzed on nanotubes similar to that used for the surface gliding assay.

### DNA scaffold–myosin preparation

DNA nanostructures were prepared based on the detailed description in our previous work (31). Single-stranded M13mp18 DNA (NEB) was mixed with fourfold excess of short stable strands (IDT), followed by 2 h annealing as previously described (41). Intact scaffolds were separated from excess staple strands using Amicon Ultra 100K cutoff spin columns (EMD Millipore). Purified scaffolds were mixed with excess labeled GIPC MIR, a mixture of 42-nt oligos with randomized sequences (blocking oligos), and 1 to 5 μM Cam in 1× AB.BSA. After 10 min of incubation at room temperature, excess streptavidin-coated magnetic beads (NEB) were added and incubated at room temperature with shaking for 10 min. The beads were washed with AB.BSA.Cam. Finally, the beads were incubated in AB.BSA.Cam containing an imaging solution of 2 mM ATP, 1 mM phosphocreatine, 0.1 mg/ml creatine phosphokinase, 45 μg/ml catalase, 25 μg/ml glucose oxidase, 1% to 2% glucose, myosin VI at 1 μM concentration, and excess elution strand for strand displacement of origami from streptavidin magnetic beads.

### DNA scaffold motility assay on single actin filaments

Motility assays were acquired at 100× magnification on an epifluorescence microscope (Nikon TE2000). Motility assays were performed using flow chambers prepared with nitrocellulose-coated coverslips (Corning 22 mm × 22 mm). First, biotinylated 488Alexa-phalloidin–stabilized actin filaments were immobilized to the inner surface of the capillary tube by BSA–biotin–neutravidin linkages. Unbound actin filaments were washed with AB.BSA. Purified GIPC–myosin VI scaffold complexes in AB.BSA.Cam + imaging reagents (2 mM ATP, 1 mM phosphocreatine, 0.1 mg/ml creatine phosphokinase, 25 μg/ml glucose oxidase, 45 μg/ml catalase, 1% glucose, and 1 μM random library 42-nt ssDNA) were added to the flow chamber. Movies of Cy5-scaffold motility on the actin filaments were obtained at 1 Hz for ≥2 min.

### Single-molecule TIRF motility assay

Flow chambers were prepared as outlined previously for surface and nanotube motility assays. BSA–biotin–neutravidin interaction was assembled on the surface similar to that described under nanotube motility assay. Biotinylated F-actin (1:9 ratio of biotin–G-actin:G-actin) was then added to the flow chamber at a concentration of 0.5 μM and incubated for 4 min. Unbound actin filaments were washed off with three flows of AB.BSA. The adaptor-MIR-GFP + Cy3-myosin VI complex or Cy3-myosin VI alone was added to the flow chamber at a concentration of 50 pM in a motility mix like that used for surface motility assay. Dual color imaging of single-molecule motility was done on a Zeiss TIRF microscope equipped with a Coherent 100 mW 488 nm and 561 nm OPSL laser and a 100× 1.4 NA oil-immersion objective with a DualView 2 for simultaneous two-channel imaging and a Photometrics QuantEM 512SC EMCCD camera for high-sensitivity single-molecule detection. Single-molecule motility was assayed at a frame rate of 10 Hz. Particle tracking of single-molecule motility data was done using the Trackmate plugin (42) in Fiji (NIH) (43, 44). Kymographs of single-molecule motility were generated using the reslice tool in Fiji. Spot intensities of Cy3-myosin VI were obtained by a line intensity scan along the kymograph in the Cy3 channel. Run lengths of adaptor–myosin VI complexes were obtained from the analysis of kymographs of motile runs in the adaptor MIR–GFP channel.

### Bimolecular FRET assay for measuring *K*_*d*_ of adaptor–motor interaction

The CBD of myosin VI (991–1294) was expressed with a C-terminal mCer (CBD-mCer). GIPC MIR was expressed with a C-terminal mCit. CBD-mCer was held constant at 30 nM, and increasing concentrations of GIPC MIR–mCit were titrated. Fluorescent spectra were collected using an excitation of 430 nm (bandpass of 4 nm) and emission from 450 to 650 nm (bandpass of 2 nm) on a fluorescence spectrophotometer (Fluoromax-4; HORIBA Scientific). To account for crossexcitation of mCit, additional spectra were collected at the same concentration of the respective MIR-mCit without CBD-mCer. These crossexcitation spectra were then subtracted from the original spectra. Every spectrum was collected three times from three separate preparations of protein. Data were collated and fit to a dose–response function using Origin (Oracle).

### Intramolecular FRET assay with myosin VI conformational sensor

A reaction mix for FRET measurement with the conformational sensor was prepared in AB.BSA.Cam buffer comprising 50 nM of the myosin VI FRET sensor protein, 2 mM ATP, and F-actin at 0.5 μM. For measurements in the presence of calcium, the reaction mix was supplemented with calcium chloride to achieve a final calcium concentration of pCa4. Measurements with adaptor proteins were performed at a saturating concentration of adaptor MIR (≥10 *K*_*d*_) by adding adaptor MIR at a final concentration of 2 μM to the reaction mix. FRET measurements were performed on a Fluoromax-4 spectrofluorometer (HORIBA Scientific) by exciting protein samples at 430 nm (mCer) with a band pass of 8 nm, and emission was monitored from 450 to 650 nm. The FRET ratio was calculated from the ratio of the emission for mCit (525 nm) to mCer (475 nm). For each experimental condition, three independent protein batches were used, and two replicates were measured for each independent experiment.

### Time-resolved anisotropy measurement with PT domain homo-FRET sensor

Time-resolved fluorescence anisotropy was performed using time-correlated single photon counting (TCSPC) and direct waveform recording methods as described previously (45). Reaction mix for time-resolved anisotropy measurements was prepared similar to that described for myosin VI conformational sensor. About 50 nM of FLASH-labeled FRET sensor was added to AB.BSA.Cam buffer containing 2 mM ATP and 0.5 μM F-actin. GIPC MIR was supplemented in the reaction mix at a saturating concentration of 2 μM. Time-resolved anisotropy measurements were performed using a DeltaPro fluorescence lifetime system (HORIBA Scientific). A 479 nm Deltadiode pulsed laser line with 515 nm LP was used for TCSPC measurements. Sequential and polarized measurements of 0°, 54.7°, and 90° were recorded for each condition. The analysis of TCSPC data was performed using the DAS6 fluorescence decay software, and the anisotropy decay was fit to a single exponential to obtain anisotropy decay time values. To describe the analysis briefly, the initial regions of the anisotropy decay were excluded because of distortions arising from instrumental response, and the later regions distributed about zero were also excluded because of high noise. The middle regions of the anisotropy curve that best captured the exponential decay were selected, and the anisotropy decay was fit to a single exponential to obtain anisotropy decay time values.

### Optical trapping experiments

#### Sample preparation

Polystyrene beads of 1 μm diameter (Polysciences: 2.5% solids [w/v]) were diluted at 1:100 dilution in AB and washed twice with AB by centrifugation at 15,000 rpm for 1 min and resuspending the bead pellet in 50 μl of AB. The beads were then conjugated with penta-HIS antibody to myosin VI CBD at a 1:10 dilution in 50 μl of AB by incubation at 4 °C with rotation for 30 min. The antibody-conjugated beads were washed twice with AB.BSA to remove unbound myosin VI antibody. Myosin VI was added to the antibody-conjugated beads in 50 μl AB.BSA.Cam at a concentration of 5 nM and incubated for 30 min. Flow chamber and single actin filaments on the coverslip surface were prepared similarly to that described under single-molecule motility assay. Beads conjugated to myosin VI were added at a 1:50 dilution in AB.BSA.Cam supplemented with an ATP regenerating mix (1 mM phosphocreatine and 0.1 mg/ml creatine phosphokinase), an oxygen-scavenging system (0.6% glucose [% w/v], 45 μg/ml catalase, 25 μg/ml glucose oxidase), and 2 mM ATP. Beads floating in solution were optically trapped and brought close to an actin filament on the surface to begin assaying myosin VI force-generation events. For GIPC condition, GIPC was added to the final mix at a saturating concentration of 2 μM to form the GIPC–myosin VI complex.

#### Optical trapping setup

The experimental setup, as reported previously (46), consists of a 1064 nm wavelength trapping laser source (Coherent, Inc) that passes through a two-axis acousto-optic deflector (catalog no.: DTD-274HA6; IntraAction Corp). The beam was expanded and steered into the microscope objective (Nikon 100×, 1.4 NA; oil immersion) using standard optical components. A detection laser (Point Source, Inc; iFLEX 2000, 50 mW, 830 nm, p-polarized) was added collinear to the trapping laser using a polarizing beam splitter cube. Intensity of the detection beam was reduced by placing a neutral density filter in its path. The intensity of the laser was adjusted such that it was less than the intensity required to trap a bead. After passing through the sample, the beams are collected by a 1.25 NA condenser. The trapping laser is blocked using a laser line filter (catalog no.: FL830-10; Thorlabs), and the back focal plane image of the detection laser is imaged onto a quadrant photodiode (catalog no.: QP50-6SD2; Pacific Silicon Sensors) with integrated amplifier circuit. The photodiode module provides three signals Vx, Vy, and Vz that represent asymmetry of light distribution on the photodiode along the *x* coordinate, *y* coordinate, and total intensity of light, respectively. The signals were captured by a field programmable gate array–based data acquisition card (catalog no.: 7833R; National Instruments). Control logic and voltage to position mapping was programmed on this hardware using custom written code in LabVIEW (National Instruments) for field programmable gate array.

#### Data acquisition

Myosin-coated beads exhibiting Brownian motion in solution were trapped using the optical trapping setup. Upon trapping a bead, the photodiode reads the bead position at 50 kHz, which was displayed on a custom LabVIEW software interface. At this point, the bead trace exhibits oscillations characteristic of Brownian motion in the trap, and its mean position indicates the center of the trap. The position of the optical trap was then gradually changed to position the bead close to an actin filament. The engagement of a motor on the bead to an actin filament produces a stroke that displaces the bead trace outside the center of the trap. Detachment of the myosin from the actin filament results in the sudden return of the bead trace back to the center of the trap. Multiple such events were recorded, and the data obtained were imported in MATLAB (Mathworks) for further analysis.

#### Data visualization

Data obtained from optical trapping experiments were analyzed with custom MATLAB software using timed bead position information from the photodiode. Bead position data acquired at 50 kHz were first filtered by applying a fourth order low pass Butterworth filter with a cutoff frequency at 10 Hz to filter out the high-frequency noise components. Filtered bead position data were then plotted against time to manually identify the presence of displacement events in the bead position data using a similar method as used (47). Upon identification, unfiltered bead position data were used to quantify and analyze stroke size, dwell times, and interarrival times of the events.

#### Data analysis

Events were manually identified based on sudden displacement of the bead trace from the center of the trap. Baseline bead position was determined by manually selecting a segment of the bead trace in which the optically trapped bead was held around 3 μm above the coverslip surface. Displaced bead position was determined by manually selecting a segment of the bead trace where the mean of the filtered bead position data was displaced from the center of the trap beyond the Brownian noise level at baseline bead position. The difference between the mean unfiltered bead position during a displacement event and the mean unfiltered baseline position gave the stroke size. The dwell time duration of a displacement event was obtained using the width of the bead trace at the peak of a displacement event. Following the identification of an event, the time duration to the next event constituted the interarrival time. Standard deviation (square root of variance) of the bead was obtained using a 200-point moving window in the bead position data and was plotted against time. A significant decrease in the standard deviation (variance clamp) was visually identified and used to estimate the stiffness of the linkage connecting the bead and the actin filament.

#### Linkage stiffness estimation

The optical trap is modeled as a simple harmonic spring with stiffness k_trap_. The stiffness of the linkage between the bead and the actin filament, k_linkage_, can be estimated by quantifying the reduction in variance of the bead position trace during myosin VI motility events in the optical trap using the equipartition theorem (48). The variance of the bead inside an optical trap is related to k_trap_ *via* the following relationship derived from the equipartition theorem:

$$\frac{1}{2}{\text{K}}_{\text{b}}\text{T}=\frac{1}{2}{\text{k}}_{\text{trap}}{\text{x}}^{2}$$

where K_b_ is the Boltzman constant, T is the ambient temperature, and x is the standard deviation of the bead position data. Thus, the lower the variance (square of the standard deviation), the higher the effective linkage stiffness. When an optically trapped bead is attached to an actin filament *via* a motor, it can be represented as a bead attached to two Hookean springs in parallel. One spring can be modeled as the optical trap itself and other as the linkage between the bead and the actin filament *via* motor(s) (Fig. 5*A*). The variance of an optically trapped bead attached to an actin filament *via* myosin(s) can therefore be represented as:

$$\frac{1}{2}{\text{K}}_{\text{b}}\text{T}=\frac{1}{2}\left({\text{k}}_{\text{trap}}+{\text{ k}}_{\text{linkage}}\right){\text{ x}}^{2}$$

To measure the effective linkage stiffness of motor(s), we first obtained the baseline stiffness of the optical trap (k_trap_) by recording the variance of the bead position when the bead is not attached to the actin filament *via* motors. Subsequently, this baseline optical trap stiffness was used to estimate the net actomyosin crossbridge stiffness (k_linkage_).

### Curve fitting to cumulative distribution functions

The raw data for dwell time were fit to a model approximating the motor’s mechanical cycle by two independent irreversible steps, which are given by the probability distribution function (PDF) of the sum of two independent exponential distributions. This model, which is given by Equation 1, has also been used previously (15) to fit the dwell times of myosin VI:

(1) $$P\left(t\text{,}k_{1}\text{,}k_{2}\right)=\frac{k_{1}k_{2}}{k_{1}-k_{2}}\left(\text{exp}\left(-k_{2}t\right)-\text{exp}\left(-k_{1}t\right)\right)$$

Where *P*(*t*,*k*_1_,*k*_2_) is the PDF with rates *k*_1_ and *k*_2_, which are the rates of two independent steps in the motor mechanical cycle. We obtained a value of *k*_1_ = 1.01 s^−1^ and *k*_2_ = 1.02 s^−1^ with GIPC as compared with *k*_1_ = 0.61 s^−1^ and *k*_2_ = 0.62 s^−1^ in the absence of GIPC MIR.

For interarrival times, events were modeled as a Poisson process with the interarrival times fitting to an exponential distribution (Equation 2), which is the defining property of a Poisson process.

(2) P(t,λ)=λ(exp(−λt))

Where *P*(*t*, *λ*) is the PDF, with *λ* as the rate parameter. This rate parameter is equal to the inverse of the mean interarrival time. We observed that the mean interarrival time for myosin VI in the presence of GIPC is 11.1 s as compared with 14.8 s in the absence of GIPC.

### DNA origami motility on keratocyte actin network

Keratocytes were derived from the scales of *Thorichthys meeki* (Firemouth cichlids) as previously described (17). Studies involving fish keratocytes were approved by the University of Minnesota Institutional Animal Care and Use Committee. Keratocytes were detergent extracted, and the actin network was stabilized with phalloidin (50 nM Alexa-488 phalloidin [Invitrogen] with 200 nM unlabeled phalloidin [MilliporeSigma]). The protocol for the six-site DNA origami preparation has been described previously. SNAP-tagged myosin VI conjugated to an oligo was patterned on the DNA origami scaffold using previously described protocols for protein assembly on DNA origami scaffolds. GIPC MIR protein was added in the final mix at a saturating concentration of 2 μM along with an ATP regenerating mix (1 mM phosphocreatine and 0.1 mg/ml creatine phosphokinase), an oxygen-scavenging system (0.6% glucose, 45 μg ml^−1^ catalase, and 25 μg ml^−1^ glucose oxidase), and 2 mM ATP. Movies of myosin VI–adaptor scaffold motility on keratocyte were acquired at 1 Hz for 5 min per field of view. Tracks from the keratocyte data were generated using the Trackmate plugin (42) in Fiji (43, 44). Further pause analysis and isolation of the tracks were performed in MATLAB using custom written code. Briefly, pauses were identified as tracks that remained within 100 nm mean square root displacement for at least four frames of video captured at one frame per second.

## Data availability

All data are contained within the article.

## Supporting information

This article contains supporting information.

## Conflict of interest

The authors declare that they have no conflicts of interest with the contents of this article.
