# Supplementary material for: Multimodal regulation of myosin VI ensemble transport by cargo adaptor protein GIPC
Source: J Biol Chem. 2022 Feb 7;298(3):101688. doi: 10.1016/j.jbc.2022.101688 (PMC8908270; doi:10.1016/j.jbc.2022.101688)
Supplement: Supplemental Figure S1 [file mmc1.pdf]

Supplemental Information

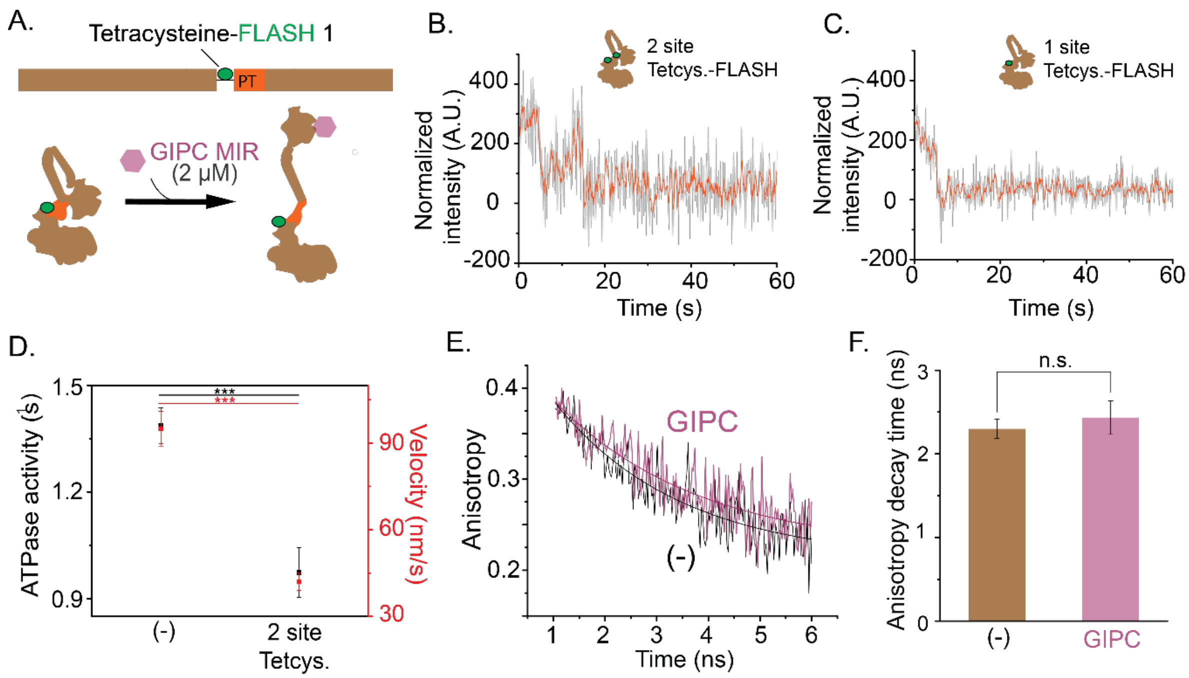

Figure S1. Characterization of labeling efficiency, motor activity and intermolecular homo FRET control for the myosin VI PT domain conformation sensor. A. Schematic representation of the construct and assay design for the single tetracysteine site PT domain homo FRET sensor. (**B and C**) Representative intensity versus time traces for single-molecule photobleaching of the PT domain homo FRET sensor to characterize FLASH-labeling efficiency of the proteins. The raw photobleaching trace is in gray and the red trace represents a 5-point averaged filtered signal of the raw data to highlight the photobleaching steps. **D.** Double Y-plot of the actin gliding velocity (black squares) and actin-activated ATPase activity (blue squares) for wild-type (WT) myosin VI compared with the 2-site tetracysteine homo FRET PT domain sensor. Error bars are S.D. Significance was computed using Students t-test. *** = P <0.001. **E.** Representative time-resolved anisotropy decay plots for the single tetracysteine site control PT domain homo-FRET sensor illustrating the decay in anisotropy over a 6 ns window. The solid line in each plot is a single-exponential fit of the anisotropy decay. **F.** Quantitation of the anisotropy decay time obtained from the single-exponential fit of the anisotropy decay. Error bars are S.D. Significance was computed using Student's t-test. n.s. = not significant.
